# The Jurisprudence of Insanity, with Especial Reference to the Case and Trial of Eugene Burt

**Published:** 1897-10

**Authors:** F. E. Daniel

**Affiliations:** Austin, Texas, Editor Texas Medical Journal, Vice-Chairman, Section of Psychology, Medico-Legal Society of New York, etc


### TEXAS MEDICAL JOURNAL.

ESTABLISHED JULY, 1885.

PUBLISHED MONTHLY.—SUBSCRIPTION $2.00 A YEAR.

VOL. XIII. AUSTIN, OCTOBER, 1897. NO. 4.

### Original Contributions.

For the Texas Medical Journal.

### THE JURISPRUDENCE OF INSANITY, WITH ESPECIAL REFERENCE TO THE CASE AND TRIAL OF EUGENE BURT.

**A Lecture Delivered by Invitation Before the Faculty and Classes of the Law School of the University of Texas, at Austin, Texas, May 29th, 1897.**

BY F. E. DANIEL, M. D., AUSTIN, TEXAS,
Editor Texas Medical Journal, Vice-Chairman, Section of Psychology, Medico-Legal Society of New York, etc,

GENTLEMEN:—Responding to the request that I address you upon some subject in Legal Medicine, I will take for my subject on this occasion, The Jurisprudence of Insanity; with Especial Reference to the Case and Trial of Eugene Burt. This furnishes an interesting and important medico-legal study, and the trial and the verdict in the Burt case illustrate, too, forcibly to my mind, the necessity for reform in the jurisprudence of insanity, as well as in certain rules and customs in the investigation and adjudication of criminal cases where insanity is set up as a defense.

In order to properly understand what I shall have to say in relation to the Burt case, it will be necessary to review,—and I will do so as briefly as is consistent with a clear understanding,—the facts in connection with the case and trial; together with certain events assumed to be facts; and also to allude to the expert testimony of certain witnesses.

The case was tried at the October term of the 26th District Court of Texas, at Austin, Texas, Hon. R. E. Brooks, presiding. An appeal was taken to the Court of Criminal Appeals, Judges Hurt, Davidson and Henderson, and came on for a rehearing on the 19th of May, as you are aware, but at this time a decision has not been rendered.

The following presentation of the case is taken from the brief of defendant submitted on appeal. Walton, Walton, and E. T. Moore, for defendant. Hon. Albert Burleson, District Attorney, representing the State in the lower court; Hon. Mann Trice, Assistant Attorney-General for the State in the higher court.

"Appellant (W. E. Burt, white, native Texan, aged 27,) was indicted in three indictments for murder of his wife and two children, little girls 4 and 2 years old. On the night of July 24th, 1896, between nine o'clock and daylight, the parties deceased disappeared. The appellant was about the house the whole of the next day, had various dealings with divers persons, and then took the train at about 11:40 o'clock, p. m. northbound, and was arrested in Chicago in about thirty days. On the fifth day after the 24th of July, the victims were found in the water in an underground cistern in the basement of the house where the family had lived. The body of the wife was wrapped up in a blanket, with a rope around it, and a handkerchief tied tightly around the neck. There was a wound in the right temple, extending down to the cheek-bone. Both the skull and cheek-bone were crushed. This wound was sufficient to produce death from hemorrhage and from shock to the brain. The handkerchief could have produced death from strangulation, and there was water enough in the cistern to have caused death by drowning. The two little girls had exact counterparts of causes for death. Their hands were crossed on the breast, and tied with wire. Their feet were also crossed at the ankles, and also tied with wire, and then wire was wound along the bodies as if to keep their clothes in place. The bodies were carried from a room upstairs, a distance of at least 100 feet, to the cistern, and therein deposited, and the top thereto securely fastened by nailing it down. There was no blood found anywhere about the premises, at any point.

"The affection of the accused towards his family was a noted and remarked fact by those who knew them.

"All the evidence which sought to connect the appellant with the killing was circumstantial. He was indicted in one count charging murder of wife, and the killing alleged to have been done with some cutting instrument; 2nd, by strangulation; and 3rd, by drowning.

"The trial came on, with plea of 'not guilty' entered. The defence was insanity; but no suggestion was made that appellant was insane at the time of the trial."

The verdict of the jury was murder in the first degree, and the penalty death.

The following facts were elicited at the trial by the lower court, the killing by defendant, and everything as detailed below, admitted by the defense, notwithstanding there were no witnesses, and all the evidence was circumstantial as to the act itself. I quote still from brief of defendant:

"On the night of July 24th, 1896, defendant and his wife were at home at half-past eight or nine o'clock, when by the nurse the younger child was delivered to him, and the elder to the wife. After the lapse of a little while he went to the dining-room, filled a bottle with milk for such younger child, went upstairs to the room where he and his wife and the children slept, leaving the wife, the elder child, and the servant in the lower rooms; this was the last of the younger child ever seen alive. After a time the servant departed, and did not return until 11 o'clock. All was quiet in the house at that time. A day or two before this he was seen coming from the stable with a grass sack in his hand which contained something. At some hour of the night, in their bed-room, the defendant killed his wife and two children. [This was assumed, admitted by defense, and was submitted as part of the hypothetical question put, later, to the expert witnesses], by striking each of them in the right temple and side of the face with a hatchet, crushing the bones of the face, and fracturing the skulls. He then tightly tied around the throat of each a handkerchief, sufficiently so to produce strangulation and suffocation; then enveloped the body of the wife, except the feet, in a blanket, and wound around the blanket ropes so as to keep the blanket in place, and the body enveloped. He tied the hands and feet of the two children with wires and other ligatures, they being in their night clothes. He then conveyed the bodies, by some means, in his arms (or by lowering them from a window, or through it, casting them out), from the upstairs room to the lower floor, to an underground cistern in the basement of the house, and cast the three bodies therein, and then nailed down the top of the cistern, which had been ripped off to admit the bodies. There was water in the cistern sufficient to submerge the bodies; the water in the cistern was in daily use by the household thereof. He took the handle of the cistern pump and secreted it. [As stated, not a sign or stain of blood was seen anywhere]. The servant returned about 11 o'clock, and slept in the house, but heard no noise, except a faint, dreamlike remembrance of hearing a child cry. The next morning at about 7 o'clock he rapped at the servant's door, awakening her, and requesting her to go to market, a thing she was not in the habit of doing. He was not seen again until the servant returned from market. On her return she took the tea-kettle, purposing to fill it with water, and in taking hold of it made a noise, when defendant said to her, 'Don't use the water from the cistern, as a cat fell in there last night.' Some questions about the wife and children arose, when he said that he had had some trouble in the night, and had sent them to San Antonio on the 5 o'clock train, but that they would be back Tuesday or Wednesday and everything would be in readiness to go keeping house at the Scott place. His breakfast being prepared, defendant gave a note to the servant to be carried to a cartman, directing him to go to the store of defendant's brothers, and procure and bring to the house some boxes; he also gave her some money to buy some nails and bring to him; all of which was done as directed; and the cartman, on bringing the boxes, was requested to return at 3 or 4 o'clock. He ate his breakfast; he sent the servant with a note to a second-hand furniture man to come and look at the furniture and other household effects. He came and looked at the effects, and asked the price wanted, and was told one-hundred and fifty dollars, but finally he agreed to take sixty-five dollars, and the trade was consummated at those figures, and the goods delivered. During the day the bloody clothing, sheets, bolsters, and pillows, and other blankets, comforts, all more or less bloody, a bloody hatchet, the hats and bonnets of the wife and miscellaneous clothing of the children (not bloody), bloody cotton from the mattress, portions of the ticking from a mattress, all bloody, were all packed in the packing boxes and nailed up, and at 4 o'clock delivered to the cartman to be conveyed to the transportation office for shipment from a fictitious person to a fictitious person in Houston, Texas. The addresses on the boxes were written in a feigned handwriting by the defendant. During the day he had various money transactions with different persons, wrote various notes, tore some up, and others were delivered to the persons to whom written. He was in or about the house the greater part of the day. In the evening the milkman came, whom defendant met at the door and said, 'This is the milkman,' got a pitcher for the milk, and told him the family had moved to 912 Rio Grande Street (there being no such street number), and that the next day he, the milkman, would find in the milk pitcher two tickets instead of one. At that time he appeared weary, as if having been hard at work, in shirt sleeves, breathing hard, and face flushed. He packed three valises and put them in the back premises of the next house during the evening, Later in the evening, towards night, he went to a hotel, ate supper, went to a barber shop and was shaved; returned to the hotel and played checkers until towards train time. Did not conceal the fact that he was on the eve of departure. To one he said he was going to Dallas; to another, San Antonio; to another, Georgetown; to another, Fort Worth. At the time named he went to the places where he had deposited his valises, obtained them, and made his way to the depot; remained in and around the depot until train time; train came in at about 11:40 p. m. Did not buy a ticket to Chicago, boarded the train, rode on it in a seat with a party whom he knew and who knew him, conversed on different subjects. He was apprehended in Chicago about thirty days thereafter, and extradited for trial, on charge of having murdered his wife and children. At the time of the murder he was out of business, without any ready means; judgment of forcible detainer had been rendered against him for the possession of the house in which he lived; process to oust him was in the hands of an officer, and the 24th of July was the last day he had permission of the owner to remain on the premises. [I will here state that he was under the impression that his bondsmen were going to give him up to the law to stand trial for forgery or embezzlement, for which he stood indicted, and his prospects for a long term of imprisonment were very strong. It is important to bear this in mind.] At no time anterior to the said July 24th, nor on that day, nor on the day subsequent thereto, did he, to many friends and acquaintances, and those with whom he transacted business, present a demeanor, appearance, habits, or conversation different to what was usual with him."

A hypothetical question, based on the foregoing, was submitted to Dr. T. D. Wooten, his two sons, Drs. Jos. S. and G. H. Wooten, Dr. M. M. Smith, Dr. R. S. Graves, city physician, Dr. J. A. Davis, late assistant physician at the lunatic asylum, and Dr. B. M. Worsham, Superintendent of the State Lunatic Asylum at Austin, witnesses for the State. They gave it as their opinion that on the night of the 24th of July, 1896, Burt was sane. These gentlemen, or some of them, at request of State's attorneys, examined the defendant in jail, taking measurements of his head, testing the reflexes, etc., with the purpose of ascertaining his mental condition at the time of the trial; a question not at issue; and they gave it as their opinion that he was sane.

It will be seen that the hypothetical question embraced none of the facts elicited from witnesses for the defense, presently to be enumerated. When a hypothetical question, embracing exclusively the facts elicited from witnesses for the defense, was put to these same witnesses, they gave it as their opinion that on the night of July 24th, Burt was insane. (See defendant's brief.) These facts were as follows; there was no controversy whatever as to these points; they were facts, established beyond question:

1. That there was insanity in the family; it was hereditary; had appeared in grandfather and other members.

2. His mother, while pregnant with him, was wild, violently insane, and had to be restrained.

3. That he was a congenital moral pervert.

4. That in childhood he was cruel, stole, lied.

5. That, as he grew into manhood, he became alienated from his brothers, his only near relatives, and without cause.

6. Subsequently he became silent, morose, stole money, embezzled money, and committed forgery when there was no need of doing so; forging checks for trifling sums, $2 and $4.

7. That he was devoted to his wife and children; had often been seen helping his wife in her household duties, even cooking; and he spent his evenings at home in preference to elsewhere, preferring the society of his family apparently to all other.

8. For the killing there was no reason nor cause that could be ascertained.

Dr. R. M. Swearingen and Dr. J. W. McLaughlin, of Austin, Dr. R. P. Talley, of Temple, Texas, an uncle of the accused, physicians of large experience in general practice; Dr. R. K. Smoot, a Presbyterian minister of Austin, were witnesses for defense. All of them had known accused more or less intimately since his childhood. These witnesses gave it as their opinion that on the night of July 24th, Burt was insane; Dr. McLaughlin qualifying his opinion by saying that he was "morally insane," a congenital "moral pervert." The State's witnesses above mentioned, when examined on the defendant's evidence, also said that he was insane on the night of the killing. (See defendant's brief.) Dr. D. R. Wallace, of Waco, Texas, summoned by defense, a physician of many years' experience in treating the insane, having been long while Superintendent of the State asylums at Austin and Terrell, generally regarded as a skilled alienist, and perhaps of all those summoned best qualified to give an opinion on the subject by further reason of his having made mental diseases a special study, gave it as his opinion that at the time of the killing defendant was of unsound mind. When asked if he was insane, answered "No, not insane, but of unsound mind."

Here I will remark that that is a distinction without a difference. All authorities agree that "insane" and "of unsound mind" are synonymous; that a person of unsound mind is insane.

Professor Fisher* says: "There is no distinction between 'insanity' and 'unsound mind.'"

So, it will be seen that Dr. Wallace, though unintentionally, gave it as his opinion that Burt was insane on the night of the killing. Nevertheless, it had all the moral effect of an adverse opinion, and was so accepted by the court.

It was also in evidence that defendant was a bright boy. He had been brought up under good moral influences, his parents being eminently respectable Christian people, and he had a happy home; yet, at an early age he showed marked depravity; would lie and steal, and was cruel to dumb creatures; nailed a living rabbit to the ground, for instance. He was an affectionate son and brother. At the time of his father's death (his father was a popular physician in Austin), in July, 1886, when this boy was sixteen years and nine months old, a marked change came over his nature and conduct. From a genial, happy member of a peaceful household, he suddenly became morose, taciturn, suspicious; held off from intercourse with the family; became alienated from his brothers,—who are exemplary citizens of Austin, and who did all in their power to assist him in his misfortunes and pecuniary troubles. They took him into their employment when he failed at all else, but he stole goods and money which he could have had for the asking; paid him out of several scrapes, and were on his bond at the time of this act, he being, as stated, under indictment for forgery or embezzlement. He regarded his brothers as his enemies, and had the belief that they had designs on his life. They had to send him away from their place of business.

There is a point here which, in my opinion, furnishes a link in the chain of presumptive evidence of insanity, which was not mentioned at the trial or brought to the attention of the experts. It is this: At the time of his father's death, when the first marked change in the boy's character was observed, he was in his seventeenth year, at the age of puberty, the period of evolution of the sexual system, when all observers agree that any tendency to insanity is apt to be developed. Puberty, pregnancy, and the puerperal state are enumerated as the critical periods of life, when any hereditary predisposition to disease or crime is apt to be developed. So well is this established that the "insanity of puberty" is enumerated as one of the marked

---

*Whittaus & Becker's System Medical Jurisprudence.

forms of the disease. In this case, with a strong hereditary predisposition, the change of habits and manners so marked would appear, taken in connection with the early evidences of a blunted sense, to be a significant and valuable diagnostic sign, which furnishes a link in the chain of progressive development of the disease. A characteristic of this form of insanity, pointed out by writers on medical jurisprudence, is, that the subject takes strong dislikes, especially to his nearest relatives.

That the jurisprudence of insanity is far behind the present status of medical science on this subject is very generally admitted; it belongs to a past age; it is not abreast of the times, and is, therefore, not adapted to the needs and requirements of a later-day civilization. This is recognized and admitted, alike by medical writers and jurists.

On this head Judge Benjamin Vaughn Abbott* in apologizing for the jurist and the lack of progress in the jurisprudence of insanity, says:

"The rude division into 'idiots' and 'lunatics' of two centuries ago survives in jurisprudence to-day. * * Jurisprudence has had no peculiar method of studying the subject, but has been accustomed to follow the course of medical science, and to accept, sometimes indeed only after long hesitation and inquiry, the results which skillful and experienced alienists have united in declaring established."

This is a very remarkable statement. What other course than that of medical science should our lawmakers follow in legislating upon a subject only understood by physicians, or, rather, better understood by physicians than by any other class of investigators? From what other source is it to be expected that jurisprudence would derive the necessary information to guide them in settling questions involving the sanity of a supposed offender?

"The English law," says Mr. Tracy Becker,† "recognizes two states of mental disease: 1st. 'Dementia Naturalis,' and 2nd, 'Dementia Adventitia,' under which head general insanity is included."

There are forty-four forms of insanity known to alienists.

It is not competent to criticise the legal points in this case, on which an appeal was taken; they are, I believe, still *sub judice*. For instance, where the burden of proof shall rest when in-

*Reference Hand-Book of Medical Sciences.
† Witthaus & Becker's System of Medical Jurispındence.

sanity is set up as defense against crime, and whether it is legal to permit a hypothetical question based on less than the whole of the evidence to be submitted to witnesses. But from the standpoint of the medical jurist, it would seem that the existing jurisprudence of insanity is not up to the standard which a better knowledge of the subject on the part of the medical profession demands; is not in accord with the later conclusions of medical science as to the nature and characteristics of the disease.

It is defective in that; (1) The defendant in a case of the kind under consideration, has not the benefit of a diagnosis by the light of modern science, because, as stated, later discoveries and conclusions of medical science are not comprehended in the existing system; the statutes have not been made to conform thereto; nor do the courts permit the use for reference of the text-books, the standard authorities; authorities are not permitted to be quoted in support of alleged insanity. (2) In that the law leaves to the determination of a jury, often of unenlightened men, metaphysical questions that baffle the ablest scientific minds, to-wit.; the existence or otherwise of insanity, the degree of impairment of free will, and the extent of responsibility to the law of a person adjudged insane by medical experts. (3) That the courts do not exercise proper discrimination in the matter of allowing medical men to pose as experts. We will discuss these objections in order.

To make clear our first proposition we will show what the popular and generally accepted conceptions of insanity are, the old pathology on which the system of jurisprudence is based, and compare it with the modern conceptions and conclusions as established by the latest authorities, and on which, I contend, a revised system should be formulated.

Prof. Charles F. Folsom* says:

"The popular idea of insanity is of wild, incoherent, or crazy conduct. If maniacal, the timid, frightened young girl, who would not hurt a fly, and the tottering, harmless old man, if confused and partly demented, are hurried off to an asylum, * * while the victim of overwhelming delusions, because he seems clear, logical, and collected, is vigorously defended against the physician's imputation of insanity, until he commits an offense against the laws, when he is fortunate if he is not treated as a criminal. It is often impossible for judges, juries, counsel, and even medical experts to wholly divest themselves

*Pepper's "System of Medicine."

of the popular notions of insanity in cases appealing strongly to the passions or prejudices of the day. Cases involving the question of responsibility for crime are decided against science and the evidence, because of certain preconceived notions of insanity which no amount of skilled opinion can controvert. Jurors and, less often, judges make up their minds what a sane man would do under given conditions, and of what an insane man is capable, judging from the facts within their own experience; and in forming their decision, it is the act itself, and not the man, diseased or otherwise, in connection with the act that chiefly governs them. * * * Strange, apparently purposeless, illogical, inconsistent action is frequently attributed to the author being insane on that subject, whereas, he may be simply acting from a strong impulse or emotion, and may be by no means insane. On the other hand, because a man knows right from wrong in the abstract, and can ordinarily behave well, the very characteristic workings of his insane mind are often siezed upon as unquestionable proof of sanity, even when admitting of no other explanation to the skilled physician than that of insanity. * * * With precisely the same degree of insanity, and the same power to control their actions, two murderers may be sentenced, the one to death for an act where the motive and method were those of the criminal, and the other to an insane asylum for killing a person under circumstances which are not explainable by sane reason."

"It is a trite but most important observation," says Buchnill* "that in the question of what constitutes insanity, the members of the two great and learned professions, law and medicine, entertain essentially different, and seemingly irreconcilable views, and that on the question of the irresponsibility of criminals who are supposed to be insane, there is a wide chasm of difference between them. To a certain extent this is true, and perhaps inevitable; and the reason for it is not hard to find; that the two professions have to regard insanity, and to deal with the insane, with different aims and purposes—the physician to prevent and cure, the main question with him being to prevent its interference with the duration and enjoyment of life. To the lawyer * * * the sole question is its existence, its degree, and its influence on the conduct; it is, with him, not a medical question, but a moral one. * * * The degree of loss of free will is a question for the jury; the fact that the will is impaired is for the expert to establish. * * * A person may be insane medically, yet not in the eye of the law. It is for the jury or experts to determine the fact of insanity; the courts to determine its effects on civil rights."

Like the shield which to one observer was golden, and to the

---

*"Insanity in its Legal Relation."

other, argent, insanity presents itself in different aspects according as it is regarded from one standpoint or another.

"Our conception of mental disease," says Prof. Fisher* "depends entirely whether we look at it from a medical or a legal standpoint."

Ray says (*ibid*):

"Insanity in medicine has to do with a prolonged departure of the individual from his natural mental state, arising from bodily disease. Insanity in law covers nothing more than the relation of the person and the particular act which is the subject of judicial investigation. The legal problem is, whether there was mental capacity and moral freedom to do, or abstain from doing, that particular act. The general meaning of insanity in law is, a permanently disordered state of the mind, produced by disease, and beyond the control of the individual."

"Very few legal mind," says Prof. B. Sachs,† "have been able to get beyond this antequated view of the relation of insanity to crime. In Germany and France the more intelligent judges have been guided by the opinions of the medical experts, but even there, they are not bound by such opinion; and it has happened, time and again, that the judge having asked for and received the opinion of the expert, has promptly set it aside, and decided the question to the contrary. * * * This right and wrong test has been the stumbling-block in the advance of legal psychiatry; and, as a matter of fact, if the test were applied to the insane (in an asylum), nine out of every ten would have to be declared sane, for the most of them are perfectly aware of the nature of the act they commit; the majority of them know that they are right or wrong according to the ordinary standards; but they are impelled, either by sudden influences, or by sudden forcible delusion, to the commission of acts which they know to be wrong, and which they, if sane, would never have committed."

"The knowledge of right and wrong‡ is not a fair criterion, as many insane men possess that knowledge well enough in the abstract. * * A man may know right from wrong, and yet not have the will-power to abstain from doing what he knows to be wrong."

Indeed. Dr. R. M. Bucke, superintendent of the largest insane asylum in Canada, with a view of determining this very question, canvassed the inmates of the asylum, and of the 1,034 inmates, found that 763 "were perfectly capable of realizing and appreciating such an act as homicide in its moral and legal relations." * * In other words, "nearly three-fourths of the

---

*Witthaus & Becker's "System Medical Jurisprudence."
†Insanity and Crime; Hamilton's System of Legal Medicine.
‡Witthaus & Becker's System of Medical Jurisprudence.

inmates were responsible and fit subjects for capital punishment as the law now exists." (See his report for 1896, just issued.)

Dr. John B. Hamilton, superintendent of State Lunatic Asylum of Illinois, at Elgin, speaking of this question in the *Journal of the American Medical Association*, says:

"The legal standard of responsibility (knowing right from wrong) given in the famous answer of the judges to the House of Lords in connection with the celebrated McNaughten case, * * which has been adopted in this country, has always, from the first, had the disapproval of competent alienists, those who of all men are best qualified to estimate the responsibility of the mentally defective. They have used every argument against it; have proved that it is a false criterion in almost every possible way, have shown clinically and pathologically its incorrectness, but have not as yet been able to thoroughly eradicate the belief in its validity from the legal mind."

He stigmatizes it as "irrational barbarism." The celebrated alienist, Dr. Morel, was seized with an irresistable impulse to throw a working man into the river, and fled from the spot to prevent doing so. Numerous cases are recorded, illustrating this fact, (not having power to resist an impulse, knowing it to be wrong); no fact is better established in the whole science of criminology. In the works on medical jurisprudence to which reference has been made, a case is related of a woman who had an impulse to kill her children, and asked to be locked up. Such cases are simply innumerable.

To many persons the sight of a sharp instrument prompts the desire or impulse to kill some one; and there are persons who do not dare to have such weapons about them for fear they may do themselves or friends harm. Burt, when arrested, asked the sheriff to take his knife, "for fear he might hurt some one." There is, indeed, a form of insanity called "reasoning insanity," in which the person understands what he is doing and the true relation of the act in its social and legal aspects. "He, however, prefers the consequences to the restless, unhappy state of mind that exists until it is done." (*Ibid.*)

It is within the last quarter of a century that the greatest advances have been made in the study of mental diseases. It is within that period that medical science has realized that insanity is a manifestation of disease of the brain, (bear in mind, though, that disease of the brain is not necessarily insanity); that the brain, the organ of the mind, is the seat of the disease; and that there can be no such thing as partial insanity. A man is insane, or he is not insane, as he may be sick or well; but it is a matter of degree. Thus, for the first time in the history of medicine has there been a scientific basis for insanity; and the study along the line suggested by this view has enabled alienists to formulate a ralional classification of the disease. In that time, too, it may be said that a new science has been born, the science of criminology, or criminal anthropology; and those cases known to alienists, and heretofore described as "border-land cases," so-called moral insanity, a condition between insanity and depravity, and barely distinguishable, if at all, are now recognized as forms of congenital madness. Gorofalo was the first to differentiate them, and to him belongs the credit of defining their characteristics. Lombroso, Gorofalo, Enrique Ferri, and others of the new school describe these as "congenital delinquents, "degenerates," "natural insane criminals;" and with painstaking care, Ferri* has pointed out the distinguishing features of each class. Lombroso and his followers have even formulated a set of physical defects or marks,—"stigmata,"— as distinctive and diagnostic; but of these I cannot here speak.

The classification made by this new school divides criminals into: (1) The madman born, (the born murderer is a born madman); (2) The homicide by occasion; (3) The homicide by passion; (4) The habital homicide. None of these concern us except the first, the natural criminal, who is always mad. He is born to kill; and, given the opportunity and the impulse, he can no more help killing than a stone can help falling when thrown into the air; he kills in obedience to an impulse for which he is not responsible, and which he cannot control.

In the congenital criminal insane (mark the distinction between "criminal insane" and "insane criminal," the one being born insane with homicidal impulses; the other being a criminal who has become insane. Flint), the most marked psychological characteristics, as pointed out by Ferri (who uses for this class the synonym, "congenital delinquent," —i. e., the victim of a hereditary predisposition to insanity, with homicidal impulses), are: moral insensibility; insensibility towards the victim, towards the sufferings of others; a cold ferocity in the execution of the crime; an apathetic impassibility after committing the crime, and even at the sight of the victim; quiet sleep after the

---

*Enrique Ferri on "Homicide," vol. 2.

deed. Such persons, whom he calls "born delinquents," are noted for their moral and physical insensibility, impassibility to their punishment, and indifference to death, often resulting in suicide. This ferocity, this indifference, says Ferri, this insensibility of the born homicide, serves as a psychological explanation of other characteristics conjoined to these. The indifference is chronic, manifesting itself in preoccupation with most trivial things, which cannot be attributed to corruption during confinement. (Note Burt's trifling conduct in prison in this connection; his putting on a mask, and charging a fee to show his face, etc.) They feel no repugnance to the idea or to the act of the homicide; they have no moral sense; they have not, in fact, any remorse concerning their offense. "To this absence of remorse must be added, stubborn denial, indifference as to escaping punishment, and the easy adaptation to prison life." "Altruistic sentiments," says the author, "such as love, family affection, etc., are not lacking in the congenital mad homicide. They are not even incapable of noble actions, but their immoral temperament renders them unstable, contradictory, and, thus, that same altruistic sentiment may find expression in their very crime." The fundamental psychological characteristic he defines thus: "An abnormal impulsiveness of action, for lack of, or owing to weak power of resistance to criminal desires; a normal man subject to such impulses can resist them." He cites also the case of Dr. Morel and other cases. The congenital mad homicide cannot thus defend himself. These facts are due, he says, to congenital weakness of development, having been arrested, and hence not apt, not educated, to resist.

Of the psycho-pathological symptoms of the congenital mad homicide, Ferri says: 1. "The deliberations of this unhappy person are due to either a slow invasion of the homicidal idea" (which he calls "homicidal obsession), or to, 2, "momentary impulse." Hence, two distinct generic types of psycho-pathological characteristics:

The first type, in which the desire to commit crime "springs from a slow and reflective process which increases from the weak or static state (obsession) until it becomes an irresistible impulse, and takes violent and dynamic form, finding vent in the criminal act. Sometimes he has a perfect cognizance of his own madness, of the act he intends to commit, and of the punishment due to it; nevertheless, this will not, cannot, deter him unless external or fortuitous causes intervene." The madman, affected by homicidal obsession, is incapable of restraining himself. A case in point is cited by this author: A man, unable to dominate the violent force impelling him to murder his wife and children, consigned himself to the police and had himself locked up.

In thc second type, the determination to homicide "proceeds from a spontaneous impulse" (as was Dr. Swearingen's opinion in the Burt case)—the "transitory mania" of the old school of psychiatry; "impulsive insanity" (homicidal) of the newer; "impulsive vertigo," without a real motive.

Perhaps the most important and significant characteristic as distinguishing the born murderer (congenital mad delinquent) from the murderer by habit or occasion, as pointed out by this advanced writer (Ferri), whose work may be taken as the exponent of the latest teachings on insanity and crime, is that, whereas the latter has always some selfish purpose or benefit in view, antisocial in its nature, murder being a means to that end, with the congenital criminal insane (of which class I regard Burt as a striking illustration), the murder is itself the end; killing to kill, impulse without motive, or "is a means to an end more often social or juridic"; that is, "as a defense of thè victims from misery or want, or a worse fate." Esquirol says that "in the delinqnent," that is, the congenital criminal insane, "murder is a means; with the common murderer it is an end, antisocial in its nature."

Still another charrcteristic of the born insane homicide (congenital delinquent), which Ferri names, is that form of insanity where the madman is possessed with the idea (obsession) to sacrifice the victim for his own good, or for the good of both self and victim. In my own mind I have not the slightest doubt but that Burt intended to complete the tragedy by suicide, but that either he was interrupted by some circumstance, or the obsession passed off before he had done so. Ferri says of the congenital mad murderer, also, that his previous conduct is often regular, when suddenly, some time before the murder, a change of life and character takes place. A more striking characteristic is his attitude during trial; his protests that he is not mad; the dissimilation of his insanity, or even simulating another form of madness from that which he suffers from; non-resistance of arrest; no attempt, or a silly one, to escape.

"The absence of any real motive," says Prof. Fisher,* "the history of hereditary taint, a neurotic disposition—seem to establish proof of mental weakness, at least approximating the confines of insanity."

Mr. Louis E. Binsse† says: "Evidence of the want of motive on the part of the accused for the perpetration of the deed is considered to be a strong corroboration of the fact of irresponsibility." Chief Justice Hornblower (State *vs.* Spencer, N. Y.) says: "I do not say that the absence of apparent motive invariably exists in cases of homicide committed by insane persons, but I say it generally is the case."

"Motiveless homicidal ideas occur to husbands and wives and parents with reference to those dearest to them, under conditions of prolonged mental strain." (Witthaus & Becker.)

"Statistics show that killing of near relatives by the congenital mad homicide occurs eight times oftener than that of any other" (*loc. cit.*).

"A crime performed without accomplices, with no plan or a silly one, for escape, and with no sane motive, is usually itself evidence of insanity." (*Ibid.*)

The last rational act Burt is known to have done on the night of the tragedy, was to take his baby from the arms of the nurse, while the mother took the elder child,—fill its bottle with milk, feed it, undress it, and get it to sleep. Within an hour or so he brained it and the others with a hatchet. Was that the act of a sane man? He packed and shipped the bloody garments and the hatchet to Houston; he went to Chicago and mingled with the people in the most public place, the Board of Exchange, meeting there acquaintances who recognized him, yet returned there again and again, knowing that a reward was offered for his arrest. Was that an effort to escape? The State asserts that there was a motive, but the best that they can offer, is "the proceeds of the sale of the furniture, $65." That is too absurd for serious consideration.

2. This brings us to a consideration of our second proposition: The most unjust and pernicious feature in our system of jurisprudence in the investigation and adjudication of cases like the one being considered, is that which leaves to a jury (if it be a jury case) the determining both of the question of the existence of insanity in the accused, the degree of impairment of

* Witthaus & Becker's System of Medical Jurisprudence.
† Theory of Criminal Responsibility.

will-power, and the responsibility of the accused to the law. Medical experts give their opinion as to the existence of insanity. Where these opinions clash, as they almost always do, it is left to the jury to decide. When it is borne in mind that the average juryman is usually a man of not a high order of intelligence,—indeed, in some cases it would appear that the juryman is selected because of his want of knowledge, ignorance being a high qualification to serve,—the absurdity of the law is apparent.

A fact is something that can be demonstrated; it is fixed; it exists. The best informed alienist cannot state it as a fact that insanity exists in a given individual; it is a matter of opinion, of judgment, the result, always, of a process of *a posteriori* reasoning; a conclusion arrived at from weighing all the evidence, and comparing the relation of facts one to another, and their bearings. The juryman has not the faculty to thus reason, because, it matters not how high or how low his native intelligence, his mind has not been trained and broadened by study. The differences of opinion between doctors when called as expert witnesses mark the differences of grade of intelligence and learning on their respective parts, as well as of attainments, and the power to reason from effect to cause. The medical man with an analytical mind, vast learning, and experience, is not liable to reach the same conclusion on a metaphysical subject, even with the same facts before him, as one of a different order of mind, or of less experience, reasoning, and deductive power. Hence, the differences between expert medical witnesses so often ridiculed by attorneys and courts, are not so illogical when looked at in the light of cultivated intelligence. It is peculiarly the mission of medical science to discover the cause of disease, and the remedy. Insanity is a disease, and, as such, is as much the exclusive province of the medical man, as is smallpox. It requires more ability to recognize occult mental disease than any other pathological condition, and yet, our system of jurisprudence relegates these intricate questions to the sole consideration and verdict of jurymen, profoundly ignorant of everything pertaining to the case. It is as illogical as to call in a layman to decide a point of diagnosis when two medical consultants have differed. "If left in doubt," says Prof. Sachs, "the jury generally decides on its own impressions; and, if in time of general excitement, usually decides against the accused whose defense is insanity." (They have no other method of deciding.)

"When, then, shall the plea of insanity be considered valid in extenuation of crime?" "The only proper answer to this question;" says Dr. Sachs,* "in the light of the present condition of psychiatry, is that no person shall be considered guilty of crime if, at the time the crime was committed, he was suffering from any form of mental disease." New York has practically made her statute accord to this. The statute says: "No act done by a person in a state of insanity can be punished as an offense." Again: "All nations agree in absolving from responsibility a person of unsound mind," says this writer (*loc cit*): In pursuance of the amended law in New York, Judge Gildersleve, in the Appellate Court of New York, charged the jury in the case of People vs. Mrs. Lubinaker presently to be referred to: "If a reasonable doubt exists as to whether the prisoner is sane or not, he is entitled to the benefit of the doubt, and to acquittal." And this is the law in most States. In Burt's case, Judge Brooks gave the jury the law to that effect.

(And here, I will ask, parenthetically, can any rational man, acquainted with the facts in Burt's case, say there was not a reasonable doubt of his sanity on the fateful night of July 24th, if indeed, his insanity were not fully established by a preponderance of medical opinion?)

I quote again from Hamilton's System of Legal Medicine":

Dr. Sachs says: "The medical expert should be called upon to state whether the accused is or was sane or insane; and if insane, he should not be held responsible for his acts."

There is a unanimity of sentiment on this head. Hence, the one important point to be established is, the existence or nonexistence of insanity in the accused when insanity is brought forward as a defense against the charge of crime.

As any departure from a physiological state, however slight, is pathological, so, given a fairly well understood standard of normal mental integrity,—sanity,—any deviation from that standard, however little, is an abnormal state, i. e., insanity; hence it will be readily understood that there may be and are innumerable grades and shades of mental unsoundness, merging the one into the other, ranging from slight alienation to violent, raving mania. No doubt there are hundreds of insane peo-

---

*Hamilton's "System of Legal Medicine."

ple amongst us, walking the streets and attending to the affairs of life, who are liable to an explosion of insanity at any moment, but who, unless, or until such explosion is brought about by the developing causes, are never suspected of any unsoundness. Dr. Oliver Wendell Holmes, in "Autocrat of the Breakfast Table," said that the worst cases of insanity are those outside of the insane asylum. Haslam, one of the first medical experts in England, declared in open court that he had never in the whole course of his life seen a sane person. And there is a growing tendency on the part of the medical profession to regard all crime as manifestations of mental alienation. Such a view, carried to its logical termination, would empty all the jails and fill all the asylums.

In this condition of affairs it will be seen that is is absolutely essential that a midway position should be determined upon, a line of distinction drawn, where responsibility ceases. But to make any such line hard and fast is an absolute impossibility; it must needs be, in the very nature of things, more or less flexible, elastic; no rule of the kind can apply to all cases, nor to all forms of insanity. Common sense, reason, and justice demand that the determination of such a question should be left to the ablest and most experienced students of mental disease.

Observe the inconsistency of the law and the courts. It is universally held that sanity is an essential requisite to crime. It is a maxim of law that an insane person cannot commit a crime. Says Judge Hurt, of the Texas Court of Criminal Appeals, before which the Burt case is now pending, awaiting his decision, in the case of Levi King *vs.* State, (see defendant's brief): "What sane mind can comprehend the possibility of a crime being committed by an insane person? If the prisoner is insane, there is no crime. If there be crime, there is no insanity. Insanity cannot excuse crime, for the fact that, if insane, there is no crime to be excused."

That is the law. It is unqualified. There is nothing said of any degree or kind of insanity; it is sufficient in the law that the party be insane. It is the province of the medical mau to prove the existence of insanity, and yet, in nearly every State —New York alone excepted, as we have seen, and that, in consequence of recent reform—it is the rule for the court to charge the jury to determine the degree of insanity, the degree of impairment of will-power, and the responsibility of even a person proven by ananimity of medical opinion to be insane. And to do this they are instructed to apply the simple antequated test of knowing right from wrong. In effect the law says, "True, Mr. Expert, you say the accused is insane; admitted; but, hold on; let *us* see *how* insane he is. Is he so insane that he does not know right from wrong? It is for you, Mr. Juryman, to determine that point." In the name of all that is consistent, how can a jury of often ignorant laymen determine such a question?

It is objected by the State (Assistant Attorney-General Mann Trice's brief, Appellate Court) that to leave the determining of the existence of insanity, the degree of will-impairment, and the responsibility of the accused, to medical experts would be tantamount to an acquittal; "insane" *ergo* "irresponsible" *ergo* "not guilty." Be it so. We maintain that it would be a wiser and more just course than that now pursued; and to an impartial mind, it would seem that a solution to this very difficult problem would be to have a Medical Court in every State, paid by the State, to whom should be left the adjudication of all points of medicine in its relation to law; just as we have courts of law to settle all legal points. Our penal system is based upon the ancient law, "an eye for an eye, and a tooth for a tooth." Vengeance appears to be the chief end; retaliation rather than justice. But that point we will not discuss here. The basis of our system was a police regulation, formulated to meet the exigencies of a barbarous, nomadic race, two thousand years ago, and is not adapted to the requirements of a latter-day civilization. Trial by jury is a relic of barbarous ages, and has degenerated. in a large number ef cases, into a travesty of law and justice. If accused of crime I would rather trust my fate to the toss-up of a penny, than to stand trial by a jury to whom is given the determining of questions so far beyond their powers of comprehension.

3. As to experts: Our 3rd proposition: The courts do not exercise proper discrimination in permitting medical men to testify as experts.

"Much of the disrepute into which hired testimony has fallen" says Prof. Hamilton* "is undoubtedly due to a kind of partnership which many men find it difficult to avoid; for the engagement of their services implies a bid for help in advancing a side by the building up of theories for the support of a more or less tenable position. * * If an expert be careless of his reputa-

*System Legal Medicine.

tion, or weak or corrupt, he will lend himself to the side of the case upon which he has been retained, and in reality he becomes a pleader."

Again, Dr. Hamilton says:

"That there is need for reform is undeniable, and that the courts do not exercise sufficient care in fixing the status of medical witnesses is equally true. The strictures of medical writers, courts and others are just, so far as the existence of demoralization goes. As the law is administered many persons can be found who are ready to arrogate knowledge and position they do not deserve. The dignified alienist of experience and reputation is comfronted by the impostor whose glib manner and bizare 'popular-science' learning sometimes impress the susceptible juryman, as does the proprietary medicine advertisement, and whose experience of medicine and its exponents is confined to the quack or cure-all. The law is largely responsible for this."

Says Dr. Sachs on this subject, (*loc. cit.*):

"Psychiatry is a very special branch of medicine. It does not constitute a part of the regular medical training in this country; yet, in some of the most important trials of recent years, any medical man has bcen accepted as an expert, and his opinion has been held to be fully as valuable as that of a man who has devoted years of study and practice to this special branch."

There is something strangely illogical, arbitrary, and inconsistent, not to say absurd, in a rule which excludes the teachings of the ablest alienists and the latest conclusions of investigators in the field of mental disease from use in trials like this; books in which are vividly drawn the clinical features of each type of insanity; and will not allow authorities to be cited as to the distinguishing characteristics of the disease; yet will allow totally inexperienced medical men, who have never in their lives treated or observed a case of insanity,—"sophomore experts," Major Walton calls them,—"to read up on authorities there is no telling how old, and then rattle off their interpretation of the text as their 'opinion.'"

It is extremely difficult for a medical witness to not share in the sympathy for or against a case, and to be uninfluenced by popular prejudice. In a case like Burt's, where the feeling against the unfortunate man was so strong and so marked, it required a brave man to run counter to the popular clamor; such man makes himself unpopular, (and unpopular means loss of patronage). In this case, one feature of rank injustice done to the prisoner was permitting men to pose as experts who had never treated or even seen a case of insanity. and were by no means expert in medicine, much less in mental pathology; and to give to their opinion equal weight with that of the alienist by profession and experience. It is not saying too much to assert that, to appearances, some of the witnesses were not altogether uninfluenced by the popular prejudice which was manifestly felt by the audience, for, to a stranger a part of the medical testimony was given in such a way as to appear to be intended to meet popular approval, and to suggest what in the language of the day is called "playing to the gallery."

A review of all the facts connected with this sad affair, and a comparison of the clinical features in the case with the descriptions of the various forms of insanity in the writings of authors cited at considerable length, forces the conviction in my mind that the defendant Burt is now, and was at the time of the murder, and had been for years, insane. His case corresponds in every detail to the description of that form of insanity which, having an hereditary origin, is developed gradually until it overpowers reason and leads to crime. That the verdict in his case was not just, however technically correct, legally, the trial may have been, was not in accord with the evidence, I firmly believe. O Justice, how many cruel wrongs are perpetrated in thy name!

Had the symptoms and all the acts of the defendant been detailed to the medical witnesses, the better-informed of them could hardly have failed to diagnose a well-marked type of the natural criminal insane degenerate of Lombrose, a born criminal of the class demonstrated by him to be always morally insane. Almost every feature in the history of the case tallies with the characteristics of the natural criminal insane with homicidal impulses, as described by most recent writers. Its counterpart can be found in many recent works, if the court had permitted them to be cited for comparison. I will go further: had counsel been permitted to read to the court and jury the clinical picture of the born insane homicide, so forcibly drawn by Ferri, and quoted above, there was not a man of ordinary intelligence, lay or professional, who was present in that room, who would not, knowing the facts in this case, have recognized Burt in the picture, a striking illustration of that type of the insane. A parallel case, to which I have referred, is that of Mrs. Lubinaker. Poor, in very bad health, a widow, eating, and feeding her three children only as she was able to earn money to buy food, pregnant, and shortly to be confined, she thought she was going to die, and the thought of her children starving prompted her to kill them. No remorse, no concern for the consequences; she realized that she had committed a crime in law, but her only idea was that they would all be better off in another world. She intended to commit suicide, and dividing the poison, "rough on rats," in four parts, one for each child and one for herself, she administered it to the children. Two of them died, but the sufferings of the other one diverted her mind from killing herself. She went for a doctor, not to save the child,—she had not thought of that,—but to relieve its sufferings; and in that way she was prevented from completing the tragedy. In the trial in the lower court she was oonvicted, being pronounced by jurors "sane." But in the higher court, expert testimony,—Dr. Allen McLane Hamilton and other equally celebrated alienists,—pronounced her insane, and of the type here being considered, and she was acquitted, This case is reported at length by Hamilton in his recent work, to which reference has been made.

It will be remembered that Ferri describes a type of the insane as above cited, who have killed their children to save them from want, and that one strong characteristic is lack of emotion, indifference even at the sight of the corpse of the victim. In this connection is recalled the stoic indifference of Burt during trial, when the bloody hatchet and the garments of his murdered innocents, stained with their young life-blood, shed by their maniac father, were exhibited to the jury and the audience. He sat as one dazed, as senseless as a stone. If he were "acting a part," as was said by some of the "experts," it was a masterpiece of acting. His stoicism would have done credit to a savage. He was said by most of the experts to be "simulating," *itself a distinguishing feature of a now well-recognized form of insanity.* The alleged experts were unable to interpret the signs, and attributed his utter insensibility to a display of "nerve," and it added to the prejudice of the populace. So flagrant was the deed, so horrible, so shocking to the finer senses, and so seemingly rational was the conduct of the unfortunate man, both before and after the deed,—what he had done,—all his acts seemed so methodical, that few would believe but that there was a deliberately planned murder,—notwithstanding no one could even conjecture a reason or motive for it,—that prejudice ran high: the people were strongly arrayed against him, and the plea of insanity was fairly laughed at. It was evident that the audience were in sympathy with the State witnesses. When damaging testimony was elicited there was a visible throb of exultation ran through the crowd. Their desire for a conviction and death sentence was so manifest that they were threatened by the court with expulsion.

There is something like cruel irony in the judge's charge to the jury, that "If you find that there was malice," etc. Malice! malice towards his devoted young wife, his sole companion in misery, who, with him, had breasted the storms of adversity without murmur! malice towards his innocent prattling babe, whom he had a short hour before lulled to sleep on his distressed bosom. One medical witness was asked on the stand: "Do you understand the workings of the human mind?" He replied, "I do." He is doubtless the only living human being thus gifted, and they should have asked him to analyse the thoughts that passed through this miserable creature's mind, the emotions that struggled in his breast that night, as he gazed upon his sleeping innocents (and his heart must have gone out to them with all the tenderness of a father's love), and realized that Grim Want, which had pursued them, "following fast and following faster," on the morrow would thrust them from beneath the roof that sheltered them, out upon the cold charity of the world; out into the streets,—beggars; he, the father, a man of some education and more or less refinement, who had been raised in comfort, if not luxury, ostracised, denied work, without money to buy bread, without friends, without resources of any kind, momentarily apprehensive of arrest and imprisonment. Ah! it would not require the genius of our gifted medical mind-reader to divine that the one thought paramount in his mind and dominant was, "What will be the fate of my loved ones, my two little daughters, when I am sent to prison, perhaps for a long term?" "Cast into this breathing world scarce half made up," mentally deficient and morally weak, heir to a propensity to evil, the heritage of a vicious ancestry, hedged in by a combination of most distressing circumstances, enough to have dethroned a reason more firmly seated, and upset a mind better balanced, is it at all strange that the impulse should have seized and overwhelmed him to end, then and there, the heartache and the unequal struggle? to kill himself and his loved ones to save them from a worse fate—kill them *because* he loved them? Say, O thou righteous judge; say, ye cheerful and willing "experts" who found him "sane"; say, ye jurors—fathers, sons, brothers—who condemned him to a felon's death; say, ye human vultures who flocked to the trial as eagles to the carcass; ye women—mothers of sons—who, neglecting home and duty, stayed to feast your morbid curiosity, to gloat over the agony of this tortured soul, who visibly exulted at every seeming evidence of guilt, and who, by your every act cried "Crucify him, crucify him!"—similarly circumstanced, what would ye have done? Oh that man should

"Glory in so rolling
On the human heart a stone."

The law should have for its object something higher, nobler than revenge. "Our system of jurisprudence," says Dr. Frederick Wines, "should not only be humane, but it should be intelligent." The protection of society, and to deter criminals and lessen crime, are the ostensible objects of capital punishment. It is a demonstrated failure. The ends can be secured by means less revolting. It is argued that, from an economic standpoint, as well as for the protection of society and future generations from the evils of hereditary transmission of criminal propensity, it would be best to exterminate this class of offenders. They are worthless to the world and to themselves, it is argued; their lives are blighted; why not hang them? To do so would be most expedient and advisable, *if we were savages;* but humanity revolts at the idea of executing an irresponsible creature; it is inhuman. The escutcheon of this free and enlightened government is already stained indelibly with the blood of too many irrational creatures, imbecile paranoiacs. In lieu of death, it is suggested that emasculation and perpetual confinement at whatever labor they may be capable of performing would be much more rational and humane, and would effectually cut off hereditary evils in time; thus affording the protection aimed at by the more brutal method in vogue.

In estimating responsibility it should be borne in mind that the warp in the physical, mental, and moral make-up of a defective antedates even his intra-uterine life. You have heard the saying, that, "To reform a drunkard, you must begin with his grandfather." The blight is in the germ—the male product that fertilizes the ovum that becomes, first, embryo, then child. In the study of Teratology we find under the microscope that the spermatozoa of syphilitics, drunkards, etc., are often deformed, distorted, diseased. These stamp the character of the father on the embryo, and shape the whole future of the resultant individual. Hence we have born into the world everything human, from the acephalous idiot, to the godlike Robert E. Lee or Gladstone. This is "the sins of the father visited upon the children even unto the third and fourth generation," and all successive generations. Such defectives are no more responsibible, morally, at least, for their character and actions than they are for being here at all. The true philosophy of the situation, as above intimated, is that, as far as it is possible to do so, such defectives should be *prevented.* A decent regard for race integrity, to say nothing of present protection, demands it; and if our marriage laws were properly amended and enforced, and the services of the surgeon were utilized as above suggested, there would, in a short while, be fewer Guiteaus, Prendergasts, and Burts to puzzle and confound our learned jurists.

I am well aware that any hope or expectation of instituting radical changes in a system so universal and so long established is utopian. But were everybody content to "accept the situation," satisfied with existing conditions, there would be no progress in any department of human activity, neither in law nor in medicine, art, science, literature, finance, or commerce. No errors would be corrected or evils eradicated. Hence, when we see that a human life so often depends upon the rules of court, based upon an antequated conception of insanity, it is not unreasonable to insist that the voice of science should be heard, and that the great truths revealed by study and research, by laborious investigation, experimentation, and compilation,—truths vital to the dearest interests of mankind,—should be utilized in medical and criminal jurisprudence. Our system needs to be recast along broader lines, and made more comprehensive; remodeled, and adapted to the changed condition of the knowledge of insanity, and the demands of an advanced civilization.

Young gentlemen,—you will soon go out into the world to practice law. Many of you will be called to the high places and will wear the ermine. When called upon to sit in judgment on the acts of some erring brother, and to pass sentence upon some fellow mortal whose lines have not been cast in pleasant places, remember,—"Vengeance is mine, saith the Lord."

In the administration of justice do not overlook the fact that to err is human, to forgive is divine; do not shut your ears to the pleadings of mercy, but let justice, be always so tempered; and in all conditions, give the unfortunate ones the benefit of the doubt.

